# Postnatal home visitation: Lessons from country programs operating at scale

**DOI:** 10.7189/jogh.08.010422

**Published:** 2018-06

**Authors:** Robert McPherson, Stephen Hodgins

**Affiliations:** 1 Save the Children, Washington, D.C., USA; 2 School of Public Health, University of Alberta, Edmonton Clinic Health Academy, Edmonton, Canada; * At the time this paper was first submitted, Dr Hodgins was employed by Save the Children, USA

## Abstract

**Background:**

Newborn mortality remains unacceptably high in many countries. Postnatal home visits (PNHVs) have been endorsed as a strategy for delivery of postnatal care (PNC) to reduce newborn mortality as well as to improve maternal outcomes. This paper reports on a review of coverage-related performance of such programs implemented at scale through government health services in Bangladesh, Ethiopia, Ghana, India, Indonesia, Malawi, Myanmar, Nepal, Pakistan, Rwanda, Sri Lanka and Uganda.

**Methods:**

We undertook a multi-country, mixed-method program review and used available survey and administrative data and key informant interviews to characterize performance of postnatal home visitation programs. In results presented in this paper, we have relied primarily on population-based surveys, notably Demographic and Health Surveys and Multi-Indicator Cluster Surveys. In addition, based on key informant interviews, we sought to understand the implementation challenges experienced delivering PNHVs, as well as responses to those challenges, in order to provide useful insights to countries to design home visitation programming when they can meet requirements for effective delivery at scale – and to identify other options when they cannot.

**Results:**

Contact coverage of PNC within 48 hours of birth following home birth (the group most prioritized in these programs) is below 10% in most of the countries reviewed; in no country does it exceed 20%. Most country programs have been unable to achieve PNHV contact coverage that would have any meaningful impact on newborn or maternal mortality. Country responses to disappointing performance have varied: some continued programming unchanged, some suspended attempts to provide PNHVs, and others modified their strategies for providing postnatal care (PNC).

**Conclusions:**

Policymakers and program managers need to consider seriously context and local feasibility when determining whether and how to use a strategy like PNHVs. At the global level, we need more than evidence of effectiveness (as determined through proof-of-concept trials) as a basis for formulating recommendations for how governments should provide services. We must also give serious attention to what can be learned from experience implementing at scale and place greater importance on feasibility of implementation in the real world.

The early postnatal period, particularly over the first hours of life, extending into the first 2-3 days of life is a period of high risk for both mother and newborn. Household practices and care provided by health workers during this period can have an important influence on outcomes. This has always been understood by professionals working in this area and is now receiving increased attention in the global maternal-newborn health community, as reflected in recommendations released by WHO in 2014 [1].

Important though this period is, it is not straightforward how best to ensure optimal practices and outcomes, particularly in settings where the overwhelming majority of births occur at home, unattended by professional health workers. We are challenged by a question that has not always been adequately considered: what strategy or strategies are likely to be most effective for improving postnatal care, faced by such constraints? This paper focuses specifically on home visitation as a strategy for improving postnatal care, recognizing that over approximately the past decade this has been the approach that has received greatest programmatic effort.

In a series of field trials that began in the mid-1990s and extended over the following decade, investigators demonstrated that in low-income country settings with high newborn mortality, a strategy relying on postnatal home visits (PNHVs) by community health workers (CHW) delivered on specific days with defined content, supported by extensive inputs, could reduce newborn mortality [2,3,4,5]. On the strength of this evidence, in 2009, WHO and UNICEF issued a joint statement recommending PNHVs for delivery of postnatal care, where appropriate [6]. For home births, the statement called for visits on days 1 and 3 and a third visit by day 7, if possible. For facility births, the first visit was to take place as soon as possible after returning home. The Joint Statement recommends specific maternal and newborn content, suitable types of health workers, and that postnatal home care by CHWs should be linked to the health system and the full continuum of care to ensure timely and appropriate care for complications. These recommendations have been reaffirmed and further developed in subsequent WHO guidance [1,7,8,9]. The 2014 WHO recommendations for postnatal care [1] call for initial postnatal care within 24 hours of birth (whether institutional or home birth), followed by contacts on day 3, between days 7 and 14 and 6 weeks after birth (without specifying where these contacts occur). These recommendations also endorse the practice of home visits over the first week after birth.

Although various proof-of-concept trials have been published [2,3,4,5], the experience of countries attempting to implement a postnatal home visitation strategy at scale has been less well captured. This paper is intended to help fill that gap.

Subsequent to the 2009 Joint Statement, many countries adopted policies to deliver postnatal home visits. Among the 75 countries included in the *Countdown to 2015* report [10], 59 have policies to deliver such home visits within one week of birth. However considerably fewer have rolled them out at scale. Anecdotal reports suggest that most governments attempting to deliver PNHVs at scale have encountered serious barriers and have not reached coverage levels needed to achieve meaningful reductions in mortality and there is documentation on problems of equitable reach [11]. These reported difficulties have raised concerns about the feasibility of providing PNHVs at scale under routine program conditions. Responding to this concern, a working group was formed in 2015, with participants from Save the Children’s Saving Newborn Lives program, WHO, UNICEF, USAID, and USAID’s Maternal Child Survival Program (MCSP) and has subsequently provided oversight to a process that was guided by the following objectives: 1) a review of country program experience implementing PNHVs at scale through government health services and 2) the development of guidance intended to inform country-level design decisions on home visitation programming as a component of a broader postnatal care strategy [12].

## METHODS

We sought to understand the structure of specific programs, their performance, and factors that influence effective implementation, drawing lessons across the various country programs. The methodology used could be characterized as a pragmatic, multi-country, mixed-methods program review. We began by using a structured process to identify study countries and then collected primary qualitative data considered together with secondary quantitative data to develop conclusions and recommendations.

Country programs were selected and studied in two phases. During phase I we consulted global experts to identify countries that have implemented postnatal home visitation programming at scale through government services. Based on their suggestions, we initially identified and screened 14 countries (6 low-income countries, 8 lower-middle-income countries) to confirm that they had implemented such programming. Respondents from each country, who included government policymakers and technical specialists from external development partners, completed a questionnaire (see Appendix 1 in **Online Supplementary Document(Online Supplementary Document)**) to describe country implementation of PNHVs. We used this information, supplemented by follow-up phone interviews and document review, to prepare profiles for the eleven countries that had implemented PNHVs at scale: Ethiopia, Ghana, India, Indonesia, Malawi, Myanmar, Nepal, Pakistan, Rwanda, Sri Lanka and Uganda. We also included Bangladesh in our review even though it had not implemented PNHVs at scale and had only just begun a one-district learning project to deliver PNHVs through government health services. The rationale for including Bangladesh was that it had carefully considered issues of feasibility of implementation of PNHVs within the local context over the past decade and only then proceeded to develop and test a public-sector implementation model – an approach that we hoped to learn from.

These twelve countries were then considered for inclusion in phase II using criteria that included evidence of implementation at scale, maturity of implementation, availability of performance data, and diversity criteria (eg, geographic location, implementation models).

**Figure 1:**
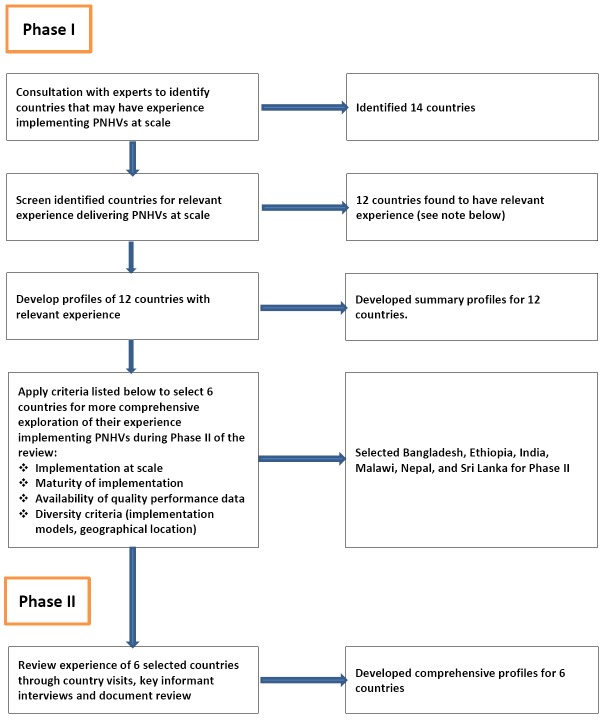
Process for selecting and screening countries during phases I and II. Note: Bangladesh was included in the review in order to better understand how it has proceeded in development of postnatal care (PNC) programming over the past decade.

Based on these criteria and potential for learning, we selected Bangladesh, Ethiopia, India, Malawi, Nepal and Sri Lanka for inclusion in phase II. During phase II we reviewed their experience implementing PNHVs through country visits, key informant interviews and document review. The process that we followed in identifying, screening and selecting countries for inclusion in our review is described in **Figure 1**.

We collected qualitative data through phone and in-person interviews (see Appendix S2 in **Online Supplementary Document(Online Supplementary Document)** for an example of a questionnaire), written questionnaires (Appendix S1 in **Online Supplementary Document(Online Supplementary Document)**) and document review to understand the development process and structure of home visitation programs and to identify key factors that influenced their performance. Authors of this paper conducted almost all interviews. We interviewed respondents that included policymakers, technical specialists from external development partners, national and district-level government health managers, health providers and clients. We took comprehensive computerized notes during interviews and then reviewed and edited within 24 hours of the interview to maximize accuracy and descriptiveness. Edited interview transcripts were then entered into a database using QDA Miner Lite V2.0 (Provalis Research, Montreal, Canada) where they were coded and analyzed.

Availability of suitable quantitative data describing performance of PNHV programming was an important challenge. Essentially all available data measuring performance at scale focused on estimating contact coverage and did not assess quality of care. The richest, most rigorously collected data were generally only available for comparatively small pilot efforts and therefore not representative of the situation as programs were progressively scaled up and institutionalized. Administrative data were generally problematic with regard to completeness and data quality. So, although we reviewed all such data, in making judgements on overall program performance, we relied most heavily on the most rigorous available secondary quantitative data to assess coverage of PNHVs for both mothers and neonates, including periodic population-based surveys, supplemented by administrative data, and special studies. Most useful were data available from Demographic and Health Surveys (DHS) and Multi-Indicator Cluster Surveys (MICS), although neither gives an altogether adequate picture of PNHV program performance. The most recent version of the DHS questionnaire (version 7) allows for disaggregated analysis of postnatal care by place, provider type and timing of PNC contact. However much less information is available in earlier surveys using version 6 and in the MICS surveys. For the countries included in this review, available DHS data are limited to studies done with version 6.

Data that governments collect on home visitation programs through routine health information systems have other limitations and can be misleading if viewed in isolation, especially when they are also used to calculate performance-based incentives for health workers who provide PNHVs. Given these constraints, to draw conclusions about overall program performance we relied primarily on data from rigorous nationally representative surveys (mainly DHS and MICS).

We used information gathered through methods described above to develop country profiles which were then shared with key stakeholders in each country for their inputs prior to finalization.

The work described in this paper did not involve any individual-level data, apart from interviews with respondents as described above. As a form of program review, it was judged by Save the Children not to be human-subjects research. The review, however, was conducted according to the principles of the Declaration of Helsinki and interviews were conducted after having obtained informed consent.

## RESULTS

In addition to the original effectiveness trials cited earlier, the literature documents pilots of home visitation packages in Bangladesh, Malawi, Nepal and Uganda [13,14]. All were designed to deliver multiple home visits during pregnancy in addition to the postnatal period. Despite intensive support, these pilots achieved only low to moderate contact coverage for early postnatal visits. Higher contact coverage was achieved for home visits during pregnancy (see [12] for more detail on these pilots).

Countries have implemented PNHVs at scale using a variety of visit schedules. Bangladesh is currently testing a model that attempts to ensure a single visit delivered within 48 hours for home births only. In contrast, Accredited Social Health Activist workers (ASHAs) in India are to make home visits on day 1 (for home deliveries) and then on days 3, 7, 14, 21, 28, and 42. Malawi is attempting to provide additional home visits to low birthweight newborns by mandating PNHVs on days 1, 3, and 8 for normal babies and, in addition, days 2 and 14 for low-birthweight babies. Indonesia has a flexible schedule for timing and location of postnatal contacts, which can take place either at home or in a health facility on days 1-2, 3-7, and 8-28.

### Contact coverage

In **Table 1** and below we provide findings on program performance (in terms of contact coverage) in delivering postnatal care particularly over the first 2 days of life. We focus on home births and PNHVs and present data from countries included in our review for which nationally representative survey data of acceptable rigor were available. All countries except Bangladesh had programs that were attempting to provide PNHVs at scale at the time of the surveys, although intensity of effort and geographic reach varied among and within countries.

**Table 1:** National, population-representative data relevant to PNHV coverage (past 2 years, unless otherwise specified)

Country (institutional delivery rate: past 5 y)	PNC coverage within 2 d of birth for mothers		For home births, timing of any PNC for the mother within 2 d of birth	PNC coverage within 2 d of birth for newborns		For home births, timing of any PNC for the newborn within 2 d of birth	Source
**Facility births**	**Home births**	**Provider type**	**Facility births**	**Home births**	**Provider type**
**Ethiopia** (16%)	48%	1%	<4 h 0.5%, 4-48 h 0.5%	–	–	–	2014 Mini DHS [15]
			physician/ midwife 0.6%, health extension worker 0.3%				
**Ghana** (73%)	93%	45%	<4 h 31%, 4-48 h 14%	25%	16%	<4 h 6.5%, 4-48 h 10%	2014 DHS [16]
			TBA 32%, nurse-midwife 7.2%, physician 1.6%, community health officer/ nurse 1.2%, other 3.5%			TBA 8.6%, physician 4.8%, nurse-midwife 2.0%, community health officer/ nurse 0.2%, other 0.8%	
**India** (79% – last 3 y)	46%	13%	–	39% (within 24 h of discharge)	13% (within 24 h of birth)	–	2014 RSOC survey [17]
**Indonesia** (63%)	89%	60%	<4 h 36%, 4-48 h 24%	53%	36%	<4 h 21%, 4-48 h 15%	2012 DHS [18]
			Nurse/midwife or village midwife 53%, TBA 6.5%, physician 0.4%, obstetrician 0.2%			Nurse/ midwife or village midwife 29%, TBA 6%, physician 0.2%, pediatrician 0.1%	
**Malawi** (89%)	82%	25%	PNC “visits” (not including care at birth): day 0: 5%, day 1: 2%, day 2: 2%, days 3-6: 2%.	88%	28%	PNC “visits” (not including care at birth): day 0: 8%, day 1: 3%, day 2: 3%, days 3-6: 2%.	2014 MICS [19]
			Location of 1st PNC visit within 1 week (among those born at home who received PNC) 82% at health facility.			Location of 1st PNC visit within 1 week (among those born at home who received PNC) 72% at health facility.	
			Physician 75%, TBA 15%, CHW 4%, community midwives 6%			Physician 66%, TBA 21%, CHW 9%, community midwives 4%	
**Myanmar** (37%)	89%	56%	<4 h 30%, 4-48 h 26%	38%	36%	<4 h 19%, 4-48 h 17%	2016 DHS [20]
			Physician/ nurse/ midwife/LHV 33%, TBA 18%, auxiliary midwife 5%, CHW 0.2%			Physician/ nurse/ midwife/ LHV-20%, TBA 12%, auxiliary midwife 3.3%, CHW 0.2%	
**Nepal** (55%)	91%	18%	PNC “visits” (not including care at birth): day 0: 1.4%, day 1%-1%, day 2 - 0.2%, days 3-6 -0.1%.	91%	17%	PNC “visits” (not including care at birth): day 0 - 2.3%, day 1 - 0.8%, day 2 - 0.6%, days 3-6 -1%.	2014 MICS [21]
			Location of 1st PNC visit within 1 week (among those born at any site who received PNC): 78% at health facility.			Location of 1st PNC visit within 1 week (among those born at any site who received PNC): 70% at health facility.	
			Physician/ nurse/ midwife 72%, auxiliary nurse-midwife 10%, health assistant/ auxiliary health worker 17%, maternal child health worker/ village health worker 2%			Physician/ nurse/ midwife 63%, auxiliary nurse-midwife 10%, health assistant/ auxiliary health worker 24%, maternal child health worker/ village health worker 3%	
**Pakistan** (48%)	85%	32%	<4 h 30%, 4-48 h 2%	58%	25%	<4 h 22%, 4-48 h 3%	2013 DHS [22]
			TBA 26%, physician /nurse/ midwife 5%, other 0.2%			Auxiliary nurse-midwife 18%, physician/ nurse/ midwife 7%, other 0.3%	
**Rwanda** (91%)	44%	31%	<4 h 24%, 4-48 h 7%	19%	19%	<4 h 13%, 4-48 h 6%	2015 DHS [23]
			Physician/ nurse/ medical assistant 27%, midwife 0.8%, CHW 2.6%			Physician/ nurse/ medical assistant 16.8%, CHW 2.2%	
**Uganda** (57%)	49%	10%	<4 h 5.5%, 4-48 h 4.5%	15%	5%	<4 h 1%, 4-48 h 4%	2011 DHS[24]
			TBA 6%, physician/nurse/midwife 4%, nurse’s aide/village health team member 0.5%, medical assistant-0.2%			TBA 1%, physician/nurse/midwife 4%, nurse’s aide/village health team member 0.1%, medical assistant-0.3%	

TBA – traditional birth attendant, CHW – community health worker, PNC – post-natal care, MICS – Multi-Indicator Cluster Surveys, d – days, h – hours

For institutional births, opportunities already exist to provide postnatal care to mothers and newborns before discharge although in most of the settings considered for this review there have been no systematic efforts to take advantage of this contact at scale. For home births, some mothers and babies access early postnatal care through a visit to a health facility. However, many cannot or do not seek facility-based postnatal care following home deliveries, leaving home visitation as a potentially attractive way to make such care available. Postnatal home visitation programs reviewed here attempted to ensure that visits were delivered by CHWs, with emphasis on an initial visit within 48 hours of birth. To varying degrees these programs prioritized home births. Reliable, independent data measuring adherence of home visitation to country-specific visit schedules were unavailable. Likewise, no data were available on the content or quality of services provided during home visits though, clearly, to have impact on health outcomes more than a mere contact is required..

We judged that data from DHS and MICS surveys generally served as a sounder basis for drawing conclusions on home visitation contact coverage than do routine administrative data sources. With the exception of India, data presented in **Table 1** are from DHS and MICS. While DHS data derived from the version 6 survey instrument cannot be disaggregated to assess PNC coverage by service site, they can be stratified by timing: within 4 hours and within 4-48 hours following birth. We assume that most PNC reported in DHS that occurred within 4 hours of birth refers to care during labor, delivery and/or immediately post-delivery (ie, part of care at childbirth), and thus does *not* represent a separate contact for provision of PNC. So from DHS survey data, we classify PNC provided within 4-48 hours following birth as *≤48hr PNC*, some of which may be delivered through home visits. DHS data also allows for PNC to be disaggregated by place of birth. Assuming a baby born at home is at least as likely to receive a home visit as one born in a health facility, *≤48hr* PNC rates for home births – as measured in DHS – can be considered to represent a maximum possible level of*≤48hr* PN home visit contact coverage, although some reported *≤48hr* PNC will have been received in a health facility. The MICS survey structures its questions and analysis in a way that allows for differentiation between care provided immediately following birth and PNC provided later in a separate contact. The MICS reporting format also allows for PNC rates to be disaggregated by service site.

From the data on postnatal care presented in **Table 1**, we see that:

- In **Ethiopia**, according to the 2014 Mini-DHS, among the 84% of women who gave birth at home, 1% reported receiving PNC within 2 days of birth. More recent data from organizations supporting the rollout of the Community-Based Newborn Care (CBNC) program estimate PNC contact coverage for mothers within 2 days of birth (home and facility combined) at 10% and 16% in two sub-regions where CBNC has been introduced [25,26]. Coverage for postnatal home visitation within 2 days of birth is probably considerably lower than 16%.
- In **Ghana**, for the 27% of newborns born at home, PNC coverage within 4-48 hours of birth was 10%. Only 0.2% of newborns born at home were reported to have received *≤48hr* PNC from community health officers or community health nurses, the two cadres primarily responsible for providing PNHVs. Most *≤48hr* PNC is provided by traditional birth attendants (TBAs) – an untrained cadre in Ghana – or is facility-based. PNHV contact coverage within 48 hours of birth from a trained provider is clearly less than 10%.
- In **India**, where 1 in 5 births occur at home, 13% of newborns born at home are reported to receive PNC within 24 hours of birth. Available data do not allow for disaggregation between PNC provided within 4 hours (ie, associated with care at childbirth) and PNC provided between 4-48 hours after birth.
- In **Indonesia**, among the 37% of births occurring at home, 15% of newborns were reported to have received PNC within 4-48 hours of birth (home plus facility). Most care was provided by midwives, the cadre responsible for conducting home visits (although they also provide PNC in facilities). These data suggest that coverage of PNHVs within 48 hours of birth is no higher than 15% for home births.
- In **Malawi**, where 1 in 10 births occur at home, 14% of newborns born at home and 11% of all newborns received a PNC visit not associated with a health check following delivery within 2 days of birth. Most of this PNC was provided at a health facility. These data suggest a PNHV coverage rate within 48 hours of birth in the low single digits.
- In **Myanmar**, 63% of births occur at home. Among newborns born at home, 17% received PNC over the period 4-48 hours after birth. Much of this care appears to be provided by TBAs, some of whom may have been trained to provide PNHVs. These data suggest that coverage of PNHVs within 48 hours of birth for newborns born at home is no higher than 17%.
- In **Nepal**, the 2014 MICS estimated the institutional birth rate at 55% and found that 4% of newborns born at home received PNC within 2 days of birth. Among the 5% of all newborns reported to have received PNC within 1 week of birth, 28% received their first PNC at home. On the basis of these data it appears that a postnatal home visit within 48 hours of birth was a rare event.
- In **Pakistan**, half of newborns were born at home, only 3% of whom received PNC within 4-48 hours of birth (at any site). Lady health workers, the government cadre nominally responsible for conducting PNHVs, are not listed as providing PNC. Coverage of PNHVs within 48 hours of birth appears to be extremely low.
- In **Rwanda**, 9 in 10 births occurred in facilities. Only 6% and 4% of babies born at home and in a health facility, respectively, received PNC within 4-48 hours of birth; only 2% and 0.2% of babies born at home and all babies, respectively, received their first PNC within 2 days of birth from a CHW. These findings suggest that coverage of PNHVs within 48 hours of birth is very low.
- In **Uganda**, among the 43% of newborns born at home, only 4% received PNC within 4-48 hours of birth. Village health team (VHT) members are nominally responsible for making home visits, but only 0.1% of babies born at home received their first PNC within 2 days of birth from a VHT member. These findings suggest that coverage of PNHVs within 48 hours of birth among home births is no higher than 4% and is most likely lower.

Considering care specifically for the mother, **Table 1** shows very similar results.

### Implications of survey findings

As stated in the introduction, published evidence suggests that effectiveness of postnatal home visitation for reducing neonatal mortality risk depends to a considerable degree on ensuring PNC visits within the first 2 days of life for babies born at home [27] (note that the published studies that demonstrated the effectiveness of PNHVs [2,3,4,5] did not use maternal mortality as an outcome measure but the interventions tested did generally include maternal content). Our analysis provides some insight into program performance on this measure, within limitations imposed by available data.

PNC provided within the first two days of life (and one strategy for its provision: home visits made during this period) should be understood as programmatically distinct from care provided immediately following childbirth. As we have categorized available DHS data, we classify “*≤48hr* PNC” as care delivered over the interval 4-48 hours after birth. In most countries presented above, coverage of*≤48hr* PNC to babies born at home is below 10%, and in no country does it exceed 20%. Coverage of*≤48hr* PNHVs is, by definition, no higher than overall coverage of*≤48hr* PNC (which also includes PNC provided in facilities).

There are no recent population-representative data that measure coverage of PNC in Sri Lanka, which has a renowned program for delivering PNHVs. According to routine health information system data, 78% of mothers received a postnatal home visit within 10 days of birth (using a denominator of total estimated births) [28]. Sri Lanka has an institutional birth rate of 99% [29], a well-adhered-to protocol requiring mother-newborn pairs to remain in the facility at least 24 hours following childbirth, and provides structured PNC before discharge. Sri Lanka is the only country in our review that appears to be achieving high coverage of PNHVs, although such contacts may not be very early.

In the data summarized above, most PNC within the first 48 hours is provided by professional health workers or health auxiliaries (and appears to occur primarily in health facilities). CHWs – the backbone of recent program efforts to provide PNHVs that were inspired by the 2009 Joint Statement – are not a significant provider of PNC in any of the reviewed surveys. Low coverage levels of home visits within 48 hours of birth provided by CHWs reinforce our finding that government efforts to deliver PNHVs reach very few mothers and newborns and therefore, as program mangers interviewed have noted, they cannot be expected to meaningfully affect newborn or maternal mortality rates.

### Response to low performance

Countries have responded in various ways to low performance of PNHV program efforts. Some have made little or no change to their programming; for example, Malawi continues to mandate home visits on days 1, 3 and 8 and is experimenting with adding management of neonatal sepsis to the content of PNHVs. Other countries have suspended efforts to systematically provide PNHVs; in response to the disappointing results of a 2012 evaluation [30] Nepal decided that female community health volunteers will no longer be expected to conduct PNHVs. Finally, some countries have modified their strategies for providing PNHVs; Rwanda reduced the schedule from 4 visits (days 1, 7, 14 and 28) to 2 (days 3 and 7-14), while in India policymakers are poised to make changes based on recent findings on barriers to effective provision of PNHVs.

### Factors influencing the performance of PNHV programs

We interviewed stakeholders from phase II countries to identify factors affecting performance of home visitation (focusing especially on contact coverage). Stakeholders noted a range of general health system issues, suggesting that a robust primary health care system may be a prerequisite for effective delivery of a PNHV strategy. Stakeholders also identified factors driving performance that are specific to PNHVs (**Table 2**).

**Table 2:** Factors that influence performance of postnatal home visits (from key informant interviews)

Factors relating to the general health system	Factors specific to PNHVs
• Government ownership and financing • Pilot-testing the model under realistic program conditions, revising the model based on evaluation findings, and only proceeding to phased scale-up if the model achieves acceptable results and demonstrates feasibility. • Adequacy of training, supervision, information collection for monitoring and evaluation, equipment and supplies, and availability of suitable referral sites for mothers and newborns with health problems. • Provisions for holding the cadre performing PNHVs accountable for services it provides.	• Adherence to a standard that mothers and newborns stay in facilities for 24 h post-delivery and be provided with high-quality PNC prior to discharge. • Schedule of PNHVs that is feasible and that may be complemented by facility-based PNC. • Positioning PNHVs as part of a life-course continuum of care rather than as a stand-alone service. • Cultivation of demand for home-based postnatal services, by clients who view the cadre providing them as competent and trustworthy, who are receptive to using government health services (including facility-based services), and who will allow health workers into their homes during the postnatal period. • Functional system for birth notification to the cadre performing PNHVs. • Availability of a cadre (to conduct PNHVs) that is qualified, motivated, has time to perform home visitation, and can access clients’ homes without undue burden. • Health worker access to transport to visit clients’ homes.

PNHV – postnatal home visits, PNC – postnatal care

## DISCUSSION

We conducted a review of postnatal home visitation programs implemented at scale under government health services, with the intention of helping countries design and strengthen locally appropriate services, particularly addressing needs in the early postnatal period. Recommendations arising from our findings are presented in **Box 1**.

Box 1 **Recommendations**

*Provide operational guidance to countries on designing context-appropriate delivery strategies:* Countries require operational guidance to help them determine how PNHVs might best fit into their mix of services and whether they have the resources to effectively deliver such services at scale and at adequate coverage. We have developed operational guidance for PNHV programming separately, as a product of this review [29].

*Encourage and facilitate local adaptation of recommendations:* Building on actual country experience documented in this paper, countries should consider developing modified schedules and approaches to implementing PNHVs that are appropriate to context and should not interpret current global PNHV guidance as inflexible. For example, some countries that have not been successful in achieving adequate coverage of PNHVs have adapted approaches to providing PNC that include: 1) increased emphasis on community-level contacts *during pregnancy* to promote key PNC practices; and 2) targeting high-risk mothers and newborns for PNHVs.

*Prioritize pre-discharge postnatal care:* Given that in many low-income countries, in recent years there have been marked increases in institutional delivery rates, countries should prioritize the provision of high-quality pre-discharge PNC to all mothers and babies born in facilities. Many countries and facilities miss the opportunity to provide this critical service. Provision of pre-discharge PNC would reduce the need for very early PNHVs – visits that are very difficult to deliver at scale in most countries.

Our findings also have implications for the process involved in developing global recommendations. Certainly, when considering new interventions or approaches, it is appropriate to begin by assessing evidence from robust proof-of-principle trials designed to allow firm conclusions with regard to causal relationships between interventions and outcomes (ie, with high internal validity). However, we argue that in many instances this is an insufficient basis for governments to adopt policy, in the absence of adequate evidence on feasibility and lessons from program experience that are relevant to specific settings where services will be delivered. This is especially true for complex interventions or service delivery strategies such as PNHVs, which are dependent on local context and available inputs.

Recommendations on complex interventions or service delivery strategies need to allow considerable flexibility and encourage context-specific judgments of relevance and feasibility. Rather than standardized templates, countries need tools and methods to support development of locally appropriate approaches tailored to the opportunities and constraints of their settings [12].

Countries have introduced PNHVs through a variety of modalities and schedules. All countries that have implemented PNHVs at scale have made serious efforts to do so. However, given the low contact coverage of PNHVs in most programs operating at scale, especially during the crucial first days following childbirth, even if such visits are delivered with good quality it is unlikely that PNHVs are achieving meaningful impact on mortality in most countries. Among countries included in the review, only Sri Lanka has documented high, sustained coverage of PNHVs at scale, although in Sri Lanka PNC within the first 48 hours after birth is systematically provided in hospitals before discharge, with follow-up home visits beginning up to 5 days later.

Among countries that have been unable to achieve acceptable coverage, some ceased efforts to deliver PNHVs while others have continued despite low performance. Still other countries have responded by modifying strategies for providing postnatal care and home visitation, illustrating the importance of learning and adaptation.

We acknowledge that program performance may have improved since the surveys that we have analyzed were conducted. Furthermore, measures of PNC coverage in these surveys are based on two-year recall, which may affect data quality and validity. And the national-level data presented here may mask better performance at the subnational level in some cases.

To date, countries implementing PNHV programming have placed primary emphasis on the PNHV visit schedule. To the extent that performance has been tracked, focus has generally been limited to whether at least one contact occurs. Increased attention to quality would benefit mothers and newborns and align with WHO’s current focus on strengthening the quality of labor and childbirth care [31]. To date available data from nationally representative surveys include essentially no information on the content or quality of PNC. This has to some extent been remedied in DHS version 7, so future surveys will provide more insight into these issues.

Effective provision of PNHVs clearly requires the support of a strong health care system. Many of the factors that stakeholders identified as crucial for the effective delivery of PNHVs also represent characteristics of a well-run primary health care system. Any strategy selected to deliver PNC should take system capacity realistically into account.

Postnatal home visitation should be understood not as a singular, stand-alone *intervention* but as a service delivery *strategy* – one of a number of ways in which PNC can be provided. A country can provide excellent PNC without necessarily making use of home visitation. What is important is that health services provide high-quality, high-coverage PNC. *Where* the service is provided is secondary. The most efficient, effective, and feasible way to provide PNC will vary among (and even within) countries.
