# Supplementary material for: Postnatal home visitation: Lessons from country programs operating at scale
Source: J Glob Health. 2018 Jun 16;8(1):010422. doi: 10.7189/jogh.08.010422 (PMC6005634; doi:10.7189/jogh.08.010422)
Supplement: Online Supplementary Document [file jogh-08-010422-s001.pdf]

Online Supplementary Document

McPherson and Hodgins. Postnatal home visitation: Lessons from country programs operating at scale

J Glob Health 2018;8:010422

Appendix S1. Screening Questionnaire for Phase 1

Instrument # 1: Policy on home births and PNC HVs in [country]

Respondent: ___________________________________

Instructions: The table below is based on the original matrix from the 2012 WHO report[1]. We would like to update the information in the 2012 report to reflect the current status. Please complete the last column as appropriate.

Question	Responses	Previous answer (from 2012)	Updated answer
Is there a national policy or strategy on skilled care at childbirth?	• Yes / no	Yes	
What is the national policy or strategy on the recommended place of childbirth? (tick one or more as appropriate)	1. Any health facility<br>2. Health facility with maternity services (labor room, maternity ward)<br>3. Home<br>4. Home and facility<br>5. No recommendation	Any health facility & Health facility with maternity services (labor room, maternity ward) (1 & 2)	
Is there a national policy on the recommended timing of discharge of mother and the baby after normal childbirth at a health facility?	• Yes / no	Yes	
If yes, after what time?	➢ Very short stay (< 6 hours)<br>➢ Short stay (6-12 hours)<br>➢ Medium stay (12-24 hours)<br>➢ Long stay (24-48 hours)<br>➢ Very long stay (>48 hours)<br>• Number of hours	short stay<br>6 hours	
Is there a national policy/ program on home births by SBA or by TBAs in [country]?	• Yes / no for SBAs<br>• Yes / no for TBAs	No for SBAs<br>?? for TBAs	
If there is a national policy on home births, please indicate the title of the SBA who should conduct home deliveries (nurse, doctor, MW or other)	• SBA<br>• non-SBA	--	
If there is a national policy on home births, what is the minimum recommended period that the SBA should observe the mother and newborn after birth at home?	• Policy on close observation after home birth? (Y/N)<br>➢ Very short observation time (< 2 hrs)<br>➢ Short observation time (2-4 hrs)<br>➢ Long observation time (> 4 hrs)<br>• Number of hours	--	

[1] Informal meeting on provision of home-based care to mother and child in the first week after birth: follow-up to the Joint WHO/UNICEF statement on home visits for the newborn child, meeting report, 8–10 February 2012. WHO 2012.

Does [country] have a national policy/strategy recommending home visits to mother and newborn in the first week after childbirth?	• PNC home visits policy/strategy exists (Y/N)<br>• Yes, for both mother and newborn<br>• Yes, for mother only<br>• Yes, for newborn only	Yes, there is a policy, for both mother and newborn	
Does the policy/strategy include home visits to pregnant mothers?	• Policy on home visits during pregnancy (Y/N)	Yes	

Instrument # 2: Description of cadres providing PNC HV services in [country]

Respondent: ___________________________________

Regarding the cadre(s) of health worker(s) / CHW(s) providing PNC home visit services …

1. **Describe the status of the cadre(s): regular government / NGO / INGO / volunteer / other:**

2. **Population covered per worker:**

3. **Employment status: full-time/part-time; paid/volunteer:**

4. **Ratio between paid CHWs and volunteers (if both are providing services):**

5. **Gender of cadre: male / female (ratio if mixed)**

6. **Residence of the cadres (to what extent are they from the localities they serve, and how does this impact on issues of language, culture and acceptance).** *Can discuss by Skype/phone as necessary.*

7. **Average duration of service of the cadres** *(please describe any effect on trust and relationship with clients).*

8. **Duration of pre-service training for cadres providing PNC home visit services.**

9. **To what extent are the cadres facility-based? What are their main service delivery modalities beyond the health facility (e.g., services from fixed outreach sites)?**

10. **What other duties do these cadres have? How difficult is it for these cadres to provide PNC home visit services given their other competing responsibilities?**

11. **Other than home visits related to maternal and newborn health (including PNC home visits), how much home visitation are the cadres actually doing?**

For the questions below, if you would like to make a written answer that would be welcomed, but if you prefer to discuss through a Skype/phone call that is fine as well.

12. **What is your perception regarding the feasibility of the cadre <u>effectively</u> carrying out his/her duties in PNC home visits as prescribed, given competing responsibilities (overall workload), capabilities/skill, and PNC HV program duties?**

13. **What is your perception regarding the strength of the community health platform in [country]? How does your assessment impact on the potential for home-based PNC services to be effectively provided there?**

Instrument # 3: Details on the PNC home visit program in [country]

Respondent: ___________________________________

Note: if there are existing documents that describe this information, please send them along with your response and feel free to quote appropriate sections below in lieu of a response.

1. **What is the schedule of expected PNC home visits?**

2. **Please describe the range of counseling/ health education content.**

3. **Please describe the dispensing functions (if any) of the cadres providing PNC home visit services.**

4. **Please describe the clinical case management functions (if any) of the cadres providing PNC home visit services, including assessment and initiation of treatment for possible sepsis, management of any complications at delivery including birth asphyxia, and assessment and management of low birth weight newborns.**

5. **Please advise regarding the existence of any documentation regarding the content of the PNC home visit program and provide any relevant documents as possible.**

Instrument # 4: Quantifying PNC home visit program performance (coverage and effectiveness) in [country]

Respondent: ___________________________________

1. **Please help to identify any and all relevant evaluations, reports and program documents, and any other available data-sets that may provide information that describes the performance (coverage and effectiveness) of the PNC home visit program in [country]?**

2. **<u>Geographic reach</u>: Please describe what part of [country]is receiving PNC home visit services. Please describe in terms of administrative units, percent of population, sub-groups, etc..**

3. **Please describe the percentage of the target population (i.e., expected births), both nationally as well as in the areas where the program is active, that is actually reached by the PNC home visit program and receives PNC HV services.**

4. **Please describe the effectiveness of the PNC home visit care that is provided (by specific intervention if possible, if data are available).**

For the questions below, if you would like to make a written answer that would be welcomed, but if you prefer to discuss through a Skype/phone call that is fine as well.

5. **Please comment on the performance and effectiveness of the PNC home visit program in [country]from your own perspective.**

6. **What are the strengths and weaknesses of the PNC home visit program in [country]?**

Appendix S2

Interview Questionnaire(s) for Phase 2

(Illustrative)

Questionnaire for national-level policymakers and program managers

General introduction

1. Introduction, objectives of review: Explain about the Global Review and its objectives, what our team is doing in [country].

2. Role of respondent regarding PNC / PN HVs: How long have you been in your current position? What is your role with regards to the provision of PNC in [country] (especially through PNHV providers)?

PNC, PN HVs and PNHV providers

3. Components of PNC programming: How would you describe [country]'s overall approach to PNC programming? What are the key components of PNC programming? Is there a targeting strategy – i.e. trying to reach high risk groups or is the strategy to do PNC home visits for all mothers? (probe: Health worker-client contacts (pre-discharge PNC, PNC HVs, PNC at facilities); BCC activities; community mobilization; health education/ counseling during pregnancy)

4. Introduction of PN HVs in programming in [country]: When were PN HVs first introduced in [country]? Pregnancy HVs? How has PNHV providers' performance of PN HVs changed over time?

5. Role of PN HVs in PNC programming: How do you view the importance of conducting PN HVs as a component of PNC programming in [country]? How does [country] balance pre-discharge PNC vs. PN HVs in terms of emphasis and importance? Why?

6. Describing the job of the PNHV provider: How can the total package of services provided by PNHV providers best be described? How do PN HVs fit in to the total efforts of PNHV providers?

PNC / PN HV policy

7. Policy regarding home-based care during pregnancy and postnatal period: What is official policy regarding the provision of postnatal care to mothers and newborns? How close are PNHV providers able to get to policy in their actual practice?

8. The effect of the 2009 WHO Joint Statement (JS) on provision of PN HV: What was the effect, if any, of the JS on [country]'s approach to providing PN HVs? At policy/operations level? On the coverage level of PN HVs? What effect, if any, has there been from the newer WHO guidelines on postnatal care of the mother and newborn?

9. Government-EDP relationships: What is the relationship between the government and partners with regards to the provision of postnatal care? What, if anything, do partners provide with regards to PNC? How important / effective is their contribution?

Performance of pregnancy and PN HVs in [country]

10. How is PN HV programming going in [country]: How well is the effort to provide PN HVs at high coverage and quality going in [country]? What are the particular strengths and weaknesses of the provision of PN HVs in [country]?

11. Targeting high risk newborns: To what extent, either in formal policy or practice, is targeting being practiced for conduct of HVs, for example in segments of the population at higher risk or for very small NBs discharged to home.

12. Feasibility of PNHV providers' performance of PN HVs: How feasible is it for PNHV providers to provide comprehensive home-based services to all pregnant and postnatal women and newborns in their catchment areas? Which aspects of their work are less feasible? More feasible? Why?

13. Quality of PNC and PNC HVs: What are your impressions of the quality of PN HV contacts with PNHV providers (i.e., what actually happens in these contacts)? What does the MoH do to assure quality of PN HVs? How is quality of PN HVs measured? Who is the appropriate person to interview about the quality of PN HVs?

Mechanics of PNC / PN HVs

14. Supervision of PNHV providers wrt PN HVs: How are PNHV providers supervised? Who supervises them? What tools are used during supervision? How effective is the supervision (note that supervision is generally poor in many LRCs)? How important is it to PNHV providers' performance, especially of PN HVs?

15. Referral systems for sick mothers, pregnant women and newborns: To what extent are PN HVs conceived as an opportunity to conduct case-finding for sick mothers and newborns? How do PNHV providers refer small / sick newborns and pregnant women with danger signs when they find them during home visits? What is the system / paper trail? When women / newborns are referred, where do they go to seek treatment? How is compliance / timeliness of compliance tracked? Is there back-referral to PNHV providers to follow-up treatment and how does that work?

Facilitators of / barriers to positive performance of PN HVs

16. Facilitators of high coverage and quality performance of HVs: In [country], what factors have contributed to positive performance of pregnancy and postnatal home visits (i.e., coverage/quality)?

17. Barriers to high coverage and quality performance of HVs: In [country], what factors have worked against positive performance of pregnancy and postnatal HVs?

18. Requirements for high performance of HVs: Based on the [country]n experience with implementing [program], what observations can you make regarding what would be required in other countries for implementation of pregnancy and postnatal home visit interventions to be effective at scale at high levels of coverage when delivered through government health services? What does it take to achieve good coverage and quality? What conditions may make that difficult or impossible?

Questionnaire for officer in charge of services in a health unit / area

1. Introduction, objectives of review: Explain about the Global Review and its objectives, what our team is doing in [country].

2. Role of respondent regarding PNC / PN HVs: How long have you been in your current position? What is your role with regards to the provision of PNC in [country] (especially through PNHV providers)?

3. Components of PNC programming: What are the key components of PNC programming in your district? (probe: Health worker-client contacts (pre-discharge PNC, PNC HVs, PNC at facilities); BCC activities; community mobilization; health education/ counseling during pregnancy)

- Is there a targeting strategy – i.e. trying to reach high risk groups or do you do PNC home visits for all mothers?

4. Policy regarding home-based care during pregnancy and postnatal period: What is official policy regarding the provision of home-based care to pregnant women? Regarding the provision of postnatal care to mothers and newborns? When should visits be made? How many? How close are PNHV providers able to get to policy in their actual practice? Why do gaps exist?

5. The effect of the JS: In 2009 WHO and UNICEF issued a statement strongly recommending that PN HVs be provided to all women including 3 visits in the first week following delivery. Did PNHV providers' approach to providing PN HVs change at all as a result of the JS? If yes, how? What effect, if any, has there been from the newer WHO guidelines on postnatal care of the mother and newborn?

6. Introduction of PN HVs: How long have PNHV providers done PN HVs in this district? Pregnancy HVs?

7. Role of PN HVs in PNC programming: How important is it to conduct PN HVs as a component of PNC programming in this district? How important are pregnancy HVs to achieve PNC goals? Why?

8. Describing the job of the PNHV provider: How can the total package of services provided by PNHV providers in this district best be described? How do PN HVs fit in to the total efforts of PNHV providers?

9. How is PN HV programming going: How well is the effort to provide PN HVs at high coverage and quality going in your area? What is working well and not so well?

10. Feasibility of PNHV providers' performance of PN HVs: How feasible is it for PNHV providers in this district to provide comprehensive home-based services to all pregnant and postnatal women and newborns in their catchment areas? What about more targeted approaches, focusing for example on very young, first-time mothers, or on very small NBs? Which aspects of their overall job are less feasible? More feasible? Why?

11. Quality of PNC and PNC HVs

- What are your impressions of the quality of PN HV contacts with PNHV providers?
- What actually happens in those contacts?
- To what extent do PNHV providers do everything they are supposed to do and do it well?
- What do you and your team do to assure quality of PN HVs?
- How is quality of PN HVs measured?

12. Facilitators of high coverage and quality performance of HVs: In this area, what factors have contributed to positive performance of pregnancy and postnatal home visits (i.e., coverage/quality)?

13. Barriers to high coverage and quality performance of HVs: In this area, what factors have worked against positive performance of pregnancy and postnatal HVs?

14. Supervision of PNHV providers wrt PN HVs:

- How are PNHV providers supervised?
- Who supervises them?
- What tools are used during supervision?
- How effective is the supervision (note that supervision is generally poor in many LRCs)?
- How important is supervision to PNHV providers' performance, especially of PN HVs?

15. Referral systems for sick mothers, pregnant women and newborns:

- To what extent are PN HVs conceived as an opportunity to conduct case-finding for sick mothers and newborns?
- How do PNHV providers refer small / sick newborns and pregnant women with danger signs when they find them during home visits?
- What is the system / paper trail?
- When women / newborns are referred, where do they go to seek treatment?
- How is compliance / timeliness of compliance tracked?
- Is there back-referral to PNHV providers to follow-up treatment and how does that work?
- Where is care provided for various types of sick newborns? Asphyxia, LBW, PSBI, hypothermia?

Questionnaire for provider of postnatal home visitation services

1. Introduction: Explain what our team is doing in [country].

2. Role of respondent regarding PNC / PN HVs: How long have you been in your current position?

3. Introduction of PN HVs: How many years have you been providing PN HVs? Pregnancy HVs?

4. Describing the job of the PNHV provider

- I would like to ask you some questions about how you spend your work time. What is the best way to talk about your work, a typical week or a typical month?
- How do you spend a typical work week / month?

- What are the major types of activities that you do? What is the best way to describe how you spend your time (probe: meetings; working in clinics; admin tasks; home visits; other)
- How much time do you spend on each activity in a week/month?
- What part of your work do you consider to be most important? The least important?
- What part of your work do you like the most? The least?
- How big a part of your work is PN and pregnancy HVs?
- How much of your time do you spend on pregnancy HVs? On PN HVs?
- How many pregnancy HVs / PN HVs do you conduct in a typical week / month?
- How do you know when a PW has delivered? What determines how many days post-delivery you visit her at home?
- How much emphasis do your supervisors place on you conducting all of your PN HVs in a timely manner? How important do you think it is to conduct PN HVs in a timely manner?

5. Policy regarding home-based care during pregnancy and postnatal period: When are you supposed to make HVs to pregnant women? HVs to mothers and newborns? How many visits are you supposed to make on which days? To what extent are you able to follow this schedule? Why do any gaps exist?

6. How is PN HV programming going: How well is your and other PNHV providers' effort to provide PN HVs at high coverage and quality going in your area? What is working well and not so well? Regarding PN HVs, which expectations are reasonable? Which ones are less/not reasonable?

7. Feasibility of PNHV providers' performance of PN HVs: How feasible is it for you to make pregnancy and PN HVs to all pregnant and postnatal women and newborns in your catchment area? Regarding other parts of your job, which parts are less feasible? More feasible? Why?

8. Quality of PNC and PNC HVs

- What do you do during a pregnancy HV?
- What do you do during a PN HV?
- To what extent is active case detection a major part of your work?

9. Facilitators of positive performance: What kinds of things make it easier for you to conduct PN HVs?

10. Barriers to positive performance: What kinds of things make it harder for you to conduct PN HVs?

11. PNHV providers' tools and equipment that they use to conduct pregnancy HVs and PN HVs

- What tools, counseling guides and equipment do you use when you conduct HVs during pregnancy and postnatal?
- How easy is it for you to get resupply / replacement?
- What handouts or other informational written materials do you distribute to pregnant or postpartum women?

- What kinds of logistical support do you receive to do your job? Bicycle, motorcycle, gasoline, phone, etc..

12. Supervision of PNHV providers wrt PN HVs

- Who supervises you?
- How is your performance of PN HVs supervised?
- How helpful is that supervision? How is it helpful / not helpful?

13. Referral systems for sick mothers, pregnant women and newborns:

- To what extent is one of the objectives of PN HVs for you to examine and assess mothers and newborns and identify sick mothers and newborns?
- What kind of newborns do you refer most often?
- How do you refer small / sick newborns and pregnant women with danger signs when they find them during home visits?
- What is the system for referring them (probe: what forms do you fill out?)
- When you refer women / newborns, where do they go to seek treatment?
- How do you track them to see if they go to seek treatment?
- Does the doctor contact you in any way after the newborn has been seen so that you can follow-up their treatment? If yes, how does that system work?

14. Forms: Can you please show me the various forms that you fill out that are related to postnatal care and pregnancy home visits? Go through the forms thoroughly; understan
